# Endoscopic Ultrasound Appearance of Jejunal Ectopic Pancreas Mimicking Metastatic Nodule in a Cancer Patient

**DOI:** 10.3390/diagnostics13040660

**Published:** 2023-02-10

**Authors:** Chien-Wei Lee, Yen-Chih Lin, Hui-Ting Hsu, Yang-Yuan Chen, Hsu-Heng Yen

**Affiliations:** 1 Division of Gastroenterology, Changhua Christian Hospital, Changhua 500, Taiwan; 2 College of Medicine, National Chung Hsing University, Taichung 400, Taiwan; 3 Department of Surgical Pathology, Changhua Christian Hospital, Changhua 500, Taiwan; 4 Department of Electrical Engineering, Chung Yuan Christian University, Taoyuan 320, Taiwan

**Keywords:** endoscopic ultrasound, ectopic pancreas, jejunal subepithelial tumor

## Abstract

The ectopic pancreas is a benign subepithelial tumor (SET) mostly found incidentally in the stomach and duodenum. Here, we present computed tomography (CT) scans and endoscopic ultrasound (EUS) images from a 71-year-old Taiwanese man newly diagnosed with colonic adenocarcinoma. CT examination revealed a mural nodule in the proximal jejunum, with good enhancement after IV contrast medium administration. Push enteroscopy was performed to localize the lesion and evaluate its nature, and a 1 cm subepithelial lesion was found. The lesion appeared hyperechoic within the submucosal layer of the bowel wall on endoscopic ultrasound examination. A tattoo was performed, and the lesion was removed during the resection of colon cancer. The histopathology confirmed the presence of pancreatic tissue inside. As far as we know, this is the first description in the literature of an endoscopic ultrasound finding of a jejunal ectopic pancreas.

A 71-year-old man with underlying hypertension and hepatitis B. He visited our gastrointestinal outpatient ward due to stool occult blood testing positive in a regular health examination. A colonoscopy revealed a fungating mass about 2 cm in size at the ascending colon. A biopsy confirms the diagnosis of colonic adenocarcinoma. In addition, an abdominal computed tomography (CT) was arranged, which reported no lymph node or distant metastatic lesions. However, a small mural nodule was found (Figure 1, arrow) in the proximal jejunum, which had good enhancement after intravenous contrast medium administration.

To evaluate the nature of the lesion pre-operatively, we performed push enteroscopy, and a 1 cm oval lesion was found in the proximal jejunum (Figure 2, arrow).

To further characterize the lesion, a mini-probe ultrasound was performed, which revealed a homogeneous-echogenic lesion involving the submucosal layer with an indistinct margin (Figure 3, arrows). A digging biopsy was taken for histological analysis of the lesion. In addition, a tattoo was injected for further surgical resection localization because the patient was planning to receive a right hemicolectomy for ascending colon cancer.

The endoscopy biopsy was negative for malignancy. Given the lesion’s feasibility and undetermined nature, the patient chose to have the jejunal tumor treated at the same time as his colon cancer via laparoscopic surgery.

The resected jejunum measured 5 cm in length and 5.5 cm in circumference. There was a tumor lesion measuring 1.5 × 1 × 0.7 cm on the mucosal surface. The histopathology revealed the presence of pancreatic tissue in the submucosal layer of the jejunum. According to Heinrich’s classification [1], Figure 4 shows type 2 pancreatic heterotopia with a mixture of pancreatic acini, ducts, and hypertrophic smooth muscle bundles in the submucosa. No nests of islet cells are identified.

The ectopic pancreas, also called the heterotopic or aberrant pancreas, is defined as pancreatic tissue that is anatomically separate from the central gland. Most of the ectopic pancreas was asymptomatic and presented as a small subepithelial tumor (SET) in the gastric antrum with a small central umbilication [2,3,4]. The ectopic pancreas could present in other gastrointestinal tracts such as the duodenum [3,5,6], jejunum [7,8,9], Meckel’s diverticulum [10], or even in the middle ear [11]. There were less than 40 cases of jejunal ectopic pancreas reported in 2022 [12]. While most of these ectopic pancreases are present in these “ectopic” locations outside the stomach, they are commonly discovered when complications occur, such as bleeding [7,13], malignancy [14,15], mass formation [8], intussusception [16], or abdominal pain [17]. The present case was found while staging a work-up of another newly diagnosed malignancy. We illustrated the usefulness of a preoperative endoscopic examination to characterize and localize the lesion for the subsequent treatment plan. A subepithelial lesion is occasionally encountered during imaging studies of the gastrointestinal tract. To differentiate the ectopic pancreas from other subepithelial lesions, there are some CT features described in the literature: (1) prominent enhancement of overlying mucosa, (2) ill-defined border, (3) location (antrum, pylorus, and duodenum), (4) long diameter/short diameter > 1.4, and (5) endoluminal growth pattern [18]. When at least 2 of these 5 criteria were used in combination, the sensitivity and specificity for diagnosing ectopic pancreas were 100% and 82.5%, respectively [5].

In addition to cross-sectional imaging, an endoscopic ultrasound (EUS) is the best tool to characterize the features (size, location, originating layer, echogenicity, and shape) of the subepithelial lesion [19]. The ectopic pancreas can develop in any layer of the GI tract wall. However, the majority involves the submucosa (15–70% of cases) and the muscularis propria (11–80%), whereas mucosal or serosal localizations are rare. GISTs, leiomyomas, and schwannomas are known to occur predominantly in the fourth wall layer (muscularis propria). The large database of the German Endoscopic Ultrasound (EUS) Registry from January 2009 to August 2013 reported the EUS character of the ectopic pancreas as heterogeneous (59%), homogeneous-hypoechoic (28.6%), and homogeneous-echogenic (7.9%) [3]. Because of the chameleon nature of the ectopic pancreas, it is not easy to make a conclusive diagnosis without the acquisition of tissue, especially in an unusual location, as in the present case. While enteroscopy is helpful in localizing and confirming the presence of a detected lesion in this patient [20], the use of endoscopic ultrasound provides additional information. The lesion was found to be homogeneous-echogenic, which was never described as far as we know in the literature. Despite the description of the EUS finding in the present case, which leads to a lower chance of a lipoma, cyst, leiomyoma, or gastrointestinal stromal tumor, we are still challenging ourselves to exclude the possibility of a metastatic lesion. Thus, we opted to tattoo the lesion for subsequent surgical resection. The diagnosis of the ectopic pancreas in the present case is made after histological confirmation.

It is tough to diagnose an incidentally detected ectopic pancreas in the jejunum. Therefore, we present a complete image study for this rare jejunal subepithelial lesion. It is the first reported EUS image of the jejunal ectopic pancreas in the literature.

## Figures and Tables

**Figure 1:**
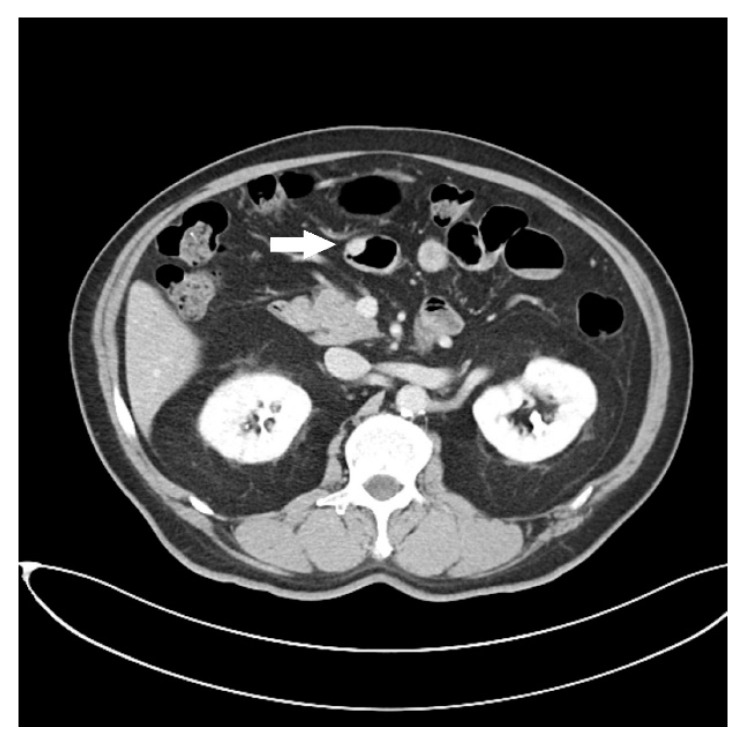
Abdominal CT finding of a small enhanced nodule in the jejunum (arrow).

**Figure 2:**
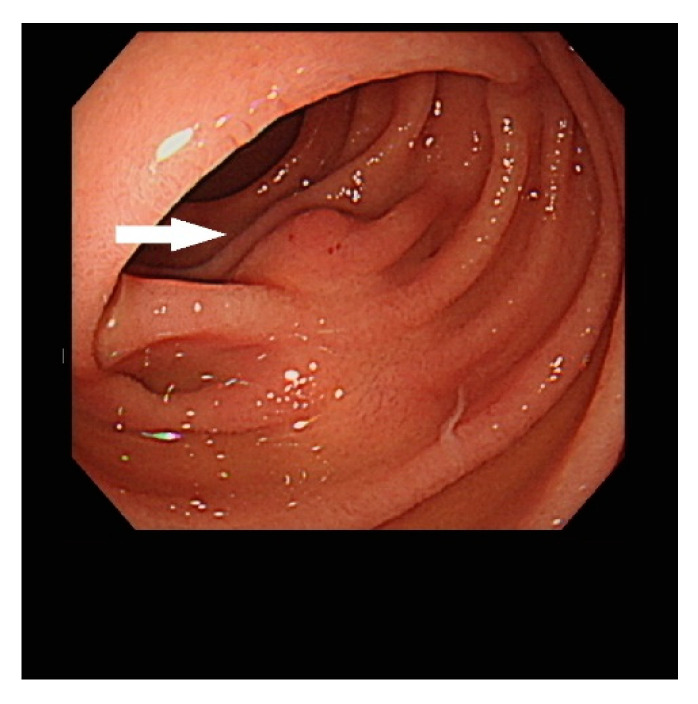
Endoscopic view of the lesion (arrow).

**Figure 3:**
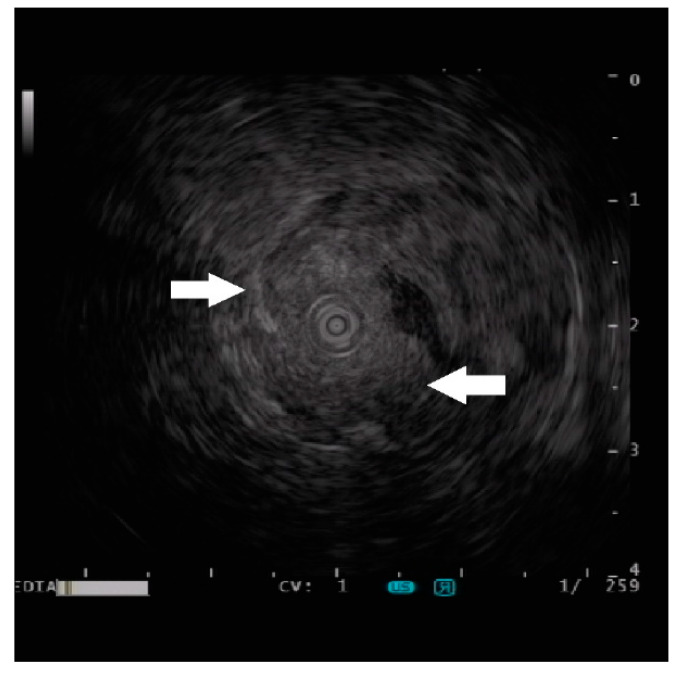
Endoscopic ultrasound image of the lesion (arrows).

**Figure 4:**
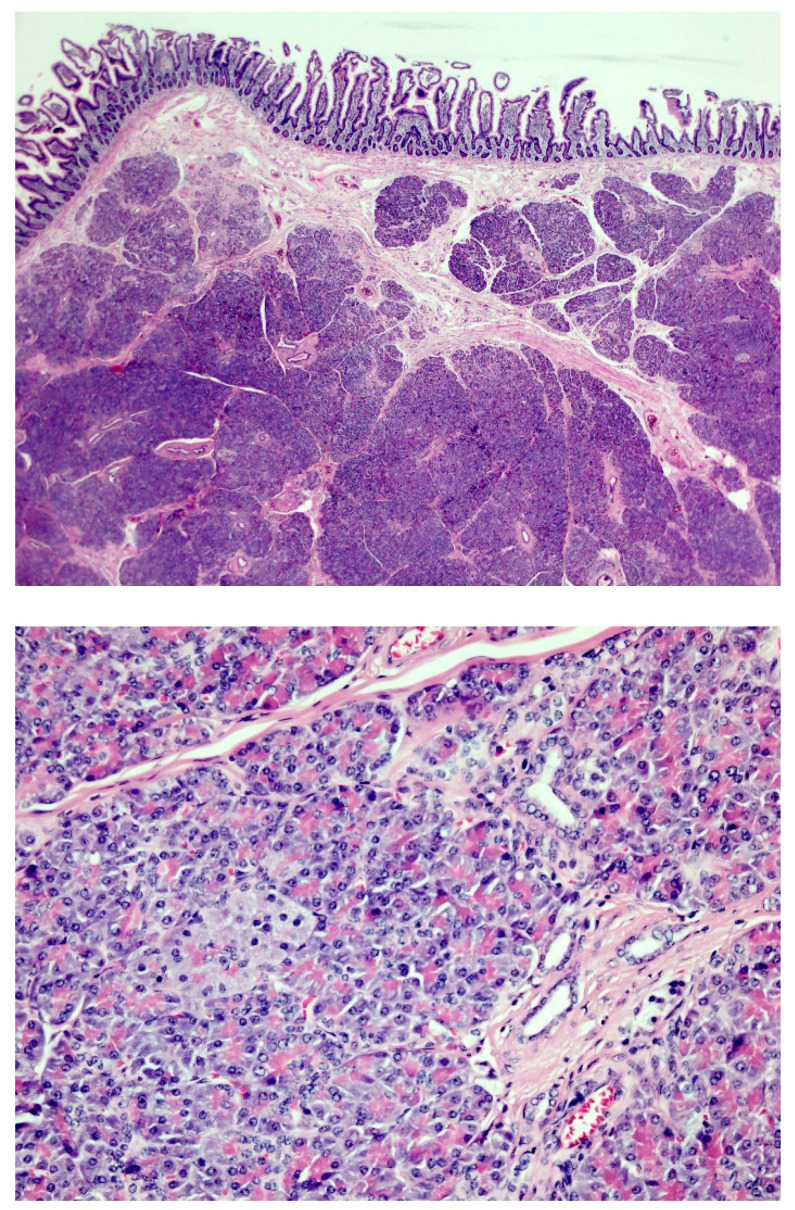
(**Upper**) hematoxylin and eosin stain, 50×; (**lower**) 100×.

## Data Availability

We did not report any data.
